# There's something afoot in the evolution of ontogenies

**DOI:** 10.1186/1471-2148-10-221

**Published:** 2010-07-22

**Authors:** Christian Peter Klingenberg

**Affiliations:** 1 Faculty of Life Sciences, University of Manchester, Michael Smith Building, Oxford Road, Manchester M13 9PT, UK

## Abstract

Allometry, the association between size and shape, has long been considered an evolutionary constraint because of its ability to channel variation in particular directions in response to evolution of size. Several recent studies, however, have demonstrated that allometries themselves can evolve. Therefore, constraints based on these allometries are not constant over long evolutionary time scales. The changes in ontogeny appear to have a clear adaptive basis, which establishes a feedback loop from adaptive change of ontogeny through the altered developmental constraints to the potential for further evolutionary change. Altogether, therefore, this new evidence underscores the tight interactions between developmental and ecological factors in the evolution of morphological traits.

## Commentary

The interplay between selective forces and the developmental processes that produce selectable variation is the focus of much attention in evolutionary biology. In a new article, Adams and Nistri [1] pursue this line of investigation by examining the evolution of growth processes in the foot of European cave salamanders. Foot morphology is particularly important in this group of salamanders because the degree of webbing between the toes has been related to their ability to cling to rocks or other substrates. Adams and Nistri use the methods of geometric morphometrics to quantify foot shape in general and to derive a measure of the degree of webbing of the feet.

For the clade of salamanders included in the study, an isometric growth trajectory, where the degree of webbing of the foot is constant over ontogeny, appears to be the ancestral condition [1]. There were at least two evolutionary changes of allometry: one lineage evolved an allometric growth pattern, where the degree of foot webbing increases with size, and a species in this lineage later reverted to an isometric mode of growth. Adams and Nistri [1] interpret the switch to allometric growth as a possible adaptation for climbing. Moreover, the ontogenetic trajectories of the different species resulted in a clear convergence from different juvenile foot morphologies toward a shared adult morphology with extensive webbing. This convergence suggests an adaptive explanation, where the common morphology corresponds to a functional optimum [1]. Overall, there appears to be a considerable degree of ontogenetic plasticity that provides opportunities for adaptive evolution.

This paper is one of a growing trend for studies at the interface of evolution and development to use morphometric methods to quantify shape [2]. Allometry and its role in evolution have long been recognized as factors that potentially can influence evolutionary processes [3-5]. In recent years, a variety of studies have provided evidence that this is indeed the case [6]. Studies like the one by Adams and Nistri [1] remind us that allometry is not necessarily a static constraint that channels variation into fixed directions in phenotypic space, but that allometry itself can evolve. Similar evolutionary flexibility of ontogenies has also been reported by recent studies in other organisms.

The evolution of allometries on a larger scale can be explored in "allometric spaces" in which the allometric pattern of each species is represented by a point and where distances between points represent the degrees of difference between allometric patterns. Gerber et al. [7] formalize this approach and illustrate it with an analysis of ontogenetic allometry in Jurassic ammonite shells, extending the approach from earlier, smaller-scale studies [8-10]. The analysis shows that there is a considerable range of allometries, which are strongly concentrated along a single axis that reflects variation in the degree to which successive whorls of the shell overlap. Moreover, the overall level of allometric variation changed remarkably little over a series of geological time zones (but there is more variation if the data are separated according to family) [7]. In this group, therefore, the variation of allometries appears to display some regular patterns across a large taxonomic scale and over geological time.

A somewhat different use of allometric spaces can be found in a comparative study of allometries in rodents by Wilson and Sánchez-Villagra [11]. This study considers ontogenetic allometry in the skull of 34 species of rodents, 17 species each from the muroid and hystricognath lineages. In the allometric space, the two lineages appear to overlap to a considerable degree, suggesting that there are no fundamental differences in allometries between them. If ecological information is added, however, a clear pattern appears: groups of species with different diets can be separated according to their cranial allometries [11]. This study indicates that ontogenetic allometries can evolve to reflect functional and ecological aspects.

These larger-scale studies reinforce the finding of Adams and Nistri [1] that allometries can evolve and that this evolution is likely to have an adaptive basis. This conclusion is interesting because allometry has been widely held to act as an evolutionary constraint--it might therefore appear counterintuitive that the constraint itself can evolve. This means that constraints affect populations at a given time and may limit or bias their potential for evolutionary change, but that these constraints don't necessarily remain constant over long evolutionary time spans [2]. The evolution of developmental systems, associated with adaptive changes in morphology, may entail evolutionary change in the developmental constraints that they cause [2].

It is likely that the ontogenetic changes in the foot morphology of salamanders [1], ammonite shell shape [7] and cranial shape in rodents [11] have an adaptive basis. Therefore, adaptive evolution of ontogenies is involved in a feed-back loop that influences the potential for further evolution by altering constraints. These studies make a timely contribution to current rethinking of the evolutionary role of constraints and their ecological implications [12].
